# Over-Expression of Allograft Inflammatory Factor-1 (AIF-1) in Patients with Rheumatoid Arthritis

**DOI:** 10.3390/biom10071064

**Published:** 2020-07-16

**Authors:** Katarzyna Piotrowska, Sylwia Słuczanowska-Głabowska, Mateusz Kurzawski, Violetta Dziedziejko, Patrycja Kopytko, Edyta Paczkowska, Dorota Rogińska, Krzysztof Safranow, Bogusław Machaliński, Andrzej Pawlik

**Affiliations:** 1 Department of Physiology, Pomeranian Medical University, 70-111 Szczecin, Poland; piot.kata@gmail.com (K.P.); sylwia@pum.edu.pl (S.S.-G.); patrycja.kopytko@pum.edu.pl (P.K.); 2 Department of Experimental and Clinical Pharmacology, Pomeranian Medical University, 70-111 Szczecin, Poland; mkurz@op.pl; 3 Department of Biochemistry and Medical Chemistry, Pomeranian Medical University, 70-111 Szczecin, Poland; viola@pum.edu.pl (V.D.); chrissaf@mp.pl (K.S.); 4 Department of General Pathology, Pomeranian Medical University, 70-111 Szczecin, Poland; edyta.paczkowska@pum.edu.pl (E.P.); doroginska@gmail.com (D.R.); machalin@pum.edu.pl (B.M.)

**Keywords:** allograft inflammatory factor-1, rheumatoid arthritis, osteoarthritis, isoforms

## Abstract

Allograft inflammatory factor-1 (AIF-1) is a cytoplasmic protein that is encoded by the *AIF1* gene. The main functions of AIF-1 are the activation of macrophages and enhancing the production of pro-inflammatory cytokines. To date, three different AIF-1 isoforms have been identified. In this study, we examined the expression of AIF-1 isoforms on the level of mRNA, and we compared the percentage of AIF-1-positive white blood cells (WBCs) in blood and AIF-1/CD68 cells in the synovial membranes in patients with rheumatoid arthritis (RA) and osteoarthritis (OA). We examined 15 patients with RA and 15 patients with OA who had previously undergone knee arthroplasty. Peripheral blood and synovial membranes (SMs) were collected from these patients during knee arthroplasty. We identified three AIF-1 mRNA expression variants in peripheral mononuclear cells (PBMCs) and SMs from patients in both groups. Spearman’s rank correlation coefficient tests showed strong, positive, and significant correlations between the three AIF-1 mRNA expression variants in PBMCs and/or SMs in patients with RA and OA. There were no statistically significant correlations for any of the AIF-1 mRNA expression variants between PBMCs and SMs in patients with RA and OA. We observed a statistically significant increased percentage of AIF-1-positive cells in patients with RA in comparison to patients with OA. The percentage of AIF-1-positive cells in the blood of patients with RA and OA was 1.35 ± 0.81% and 0.71 ± 0.25% (*p* < 0.01), respectively, whereas the percentage of AIF-1/CD68-positive WBC cells in the SMs was 24.05 ± 7.17% and 4.78 ± 1.52% (*p* < 0.001), respectively. In conclusion, three AIF-1 mRNA expression variants occurred in PBMCs and SM cells in patients with RA and OA. The AIF-1 mRNA expression levels of the variants correlated with each other in PBMCs and SM cells, but there were no statistically significant correlations for AIF-1 mRNA expression variants between PBMCs and SM cells in patients with RA and OA. Both in the blood and SMs, we observed an increased percentage of AIF-1-positive cells in patients with RA in comparison to patients with OA. The above results suggested that AIF-1 was the cytokine involved in the pathogenesis of RA. The precise knowledge of the role of AIF-1 in RA pathogenesis and the development of inflammatory response requires further investigations.

## 1. Introduction

Rheumatoid arthritis (RA) is a chronic autoimmune disease, resulting in joint damage and functional disability. Joint destruction results from the action of activated immune cells in the joint tissue, with inflammatory factors causing migration, activation, and intensive secretion of immune cells [1]. Osteoarthritis (OA) commonly develops in the absence of joint degeneration [2], although some synovial inflammation is noted [3].

Allograft inflammatory factor-1 (AIF-1) has been first described in macrophages in rat cardiac allografts upon rejection [4]. Similarly, AIF-1 expression has been further demonstrated in neutrophils, T-cells, microglia, fibroblasts, and vascular smooth muscle cells (VSMCs), where AIF-1 is implicated in cell activation, phagocytosis, proliferation, migration, and cytokine production [5,6,7,8,9,10,11,12]. AIF-1 has also been described in different cancer cell lines, where it is responsible for cell proliferation and migration in some cancer patients [13,14]. AIF-1 is the molecule involved in the pathogenesis of diseases with chronic inflammatory response, especially those involving macrophages, such as rheumatoid arthritis. AIF-1 stimulates macrophages to produce various cytokines involved in inflammatory status in RA. AIF-1 is one of the factors responsible for inflammation in RA [15,16]. In this disease, AIF-1 is detected in peripheral blood mononuclear cells (PBMCs), monocytes, and macrophages in synovial membranes (SMs) [17]. Cultured RA fibroblast-like synoviocytes in the presence of AIF-1 have been induced to produce cytokines [15]. AIF-1 has also caused increased cell viability and prevented apoptosis in RA synoviocytes [18].

To date, three different AIF-1 isoforms have been identified [19]. Isoform variant 1 (v.1) and v.3 have been detected in patients with breast cancer [14]. According to the National Center for Biotechnology Information (NCBI) gene database, mRNA sequences for v.1 and v.4 code for the same protein called isoform v1, and v.3 code for the protein isoform 3 [20]. AIF-1 v.1 expression correlates with anthropometric parameters in patients with breast cancer, and v.3 is the longest isoform, which is also called the “wild type”. The protein contains an EF-hand domain implicated in calcium-binding [8,14]. In patients with breast cancer, the expression of AIF-1 isoforms (variants) has been observed in breast adipose tissue monocytes and M1 macrophages. Studies have suggested that v.1, v.2, and v.3 are present in breast cancer and systemic sclerosis, but they “behave” differently and possibly play different roles in disease progression [14,21]. Previous studies have shown increased expression of AIF-1 protein or AIF-1-positive cells in RA patients. Kimura et al. detected increased expression of AIF-1 in synovial membranes and synovial fluid of RA patients. Additionally, AIF-1 increased the proliferation of cultured synovial cells and enhanced the synthesis of interleukin (IL)-6 [15]. Harney et al. demonstrated increased expression of AIF-1 in macrophages from RA patients [22]. In our previous study, we indicated an increased number of AIF-1-positive cells in the blood and SMs from patients with RA [17]. So far, the AIF-1 expression at the mRNA level in RA patients has not been investigated.

It is not known if AIF-1 plays an important role in initiating the inflammatory response in patients with RA or is produced under the influence of pro-inflammatory mediators released in patients with RA. In this study, we examined the expression of AIF-1 isoforms on the level of mRNA, and we compared the percentage of AIF-1-positive cells in patients with RA and OA.

## 2. Materials and Methods

### 2.1. Patients

We examined 15 patients with RA (12 female, 3 male, mean age 64.3 ± 8.4, mean erythrocyte sedimentation rate (ESR) values 18.5 ± 5.8, mean C- reactive protein (CRP) values 22.7 ± 4.6 mg/dL), who were diagnosed according to the American College of Rheumatology criteria, and 15 patients with OA RA (11 female, 4 male, mean age 67.1 ±7.8, mean ESR values 6.5 ± 2.3, mean CRP values 4.8 ± 1.9 mg/dL), who had undergone knee arthroplasty. Peripheral blood and SMs were collected during surgery. The patients were recruited in accordance with the principles of the Declaration of Helsinki. The study was approved by the Ethics Committee of Pomeranian Medical University, Szczecin, Poland (KB-0012/39/17) and the written informed consent was obtained from all subjects.

### 2.2. Quantitative Real-Time Polymerase Chain Reaction

Total RNA was extracted from 50 mg SM samples using the TRIzol-based RiboPure RNA Purification Kit (Life Technologies, Carlsbad, CA, USA), with an initial rotor-stator homogenization step. For RNA isolation from fresh whole blood samples, a RiboPure Blood Kit was used. RNA was extracted from 0.5 mL of the white blood cell (WBC)-rich layer after blood fractionation by centrifugation at 1500–2000 g for 10 min at room temperature. All samples were digested with DNAase. RNA quantity and purity were measured using a NanoDrop ND-1000 spectrophotometer (NanoDrop Technologies, Wilmington, DE, USA), and samples were stored at −80 °C until required.

### 2.3. Real-Time PCR Reaction

cDNA was prepared from 250 ng total RNA in a 20 μL reaction volume using the RevertAid Premium First Strand cDNA Synthesis Kit (Fermentas, Thermo Scientific, Waltham, MA, USA) and oligo-dT primers, according to the manufacturer’s instructions. The quantitative expression of three *AIF-1* alternative transcripts (v.1: NM_032955.2, v.3: NM_001623.4, and v.4: NM_001318970.2) was measured using transcript-specific pre-designed TaqMan assays, validated by the supplier (Hs00897091_g1, Hs00357551_g1, and Hs00894881_gH; Life Technologies, Carlsbad, CA, USA). Three transcripts encoded two AIF-1 protein isoforms: v.1 and v.4, which encoded the same protein isoform, while the protein encoded by v.3 had a longer N-terminus. The following housekeeping reference genes were used: G*APDH* (glyceraldehyde-3-phosphate dehydrogenase), *GUSB* (beta-glucuronidase), and *HPRT1* (hypoxanthine phosphoribosyltransferase 1). qRT-PCR was performed in a total volume of 15 µL in a 7500 Fast Real-Time PCR System (Applied Biosystems, Foster City, CA, USA) using the TaqMan GE Master Mix (Life Technologies). Each sample was analyzed simultaneously in two technical replicates, and the mean C_T_ values were used for further analysis. The relative gene expression was analyzed by 7500 Fast Real-Time PCR System Software version 2.04 (Applied Biosystems), using the ΔC_T_ relative quantification method normalized to the *GUSB, GAPDH,* and *HPRT1* genes. 

### 2.4. Stimulation of Peripheral Blood Mononuclear Cells (PBMCs)

Peripheral blood (PB) samples (*n* = 9) were lysed twice using BD Pharm Lyse Buffer (BD Bioscience) at room temperature for 10 min and subsequently washed in phosphate-buffered saline (PBS) with 2% fetal bovine serum (FBS; Sigma—Merck, Burlington, MA, USA) to yield total nucleated cells (TNCs). After lysis, cells were counted and prepared for stimulation.

Cells were seeded in a 10^6^ cells/35-mm dish in DMEM medium and incubated with IL-6 (100 ng/mL Merck, Warsaw, Poland), IL-21 (50 ng/mL, Bio-techne, Minneapolis, MN, USA), and IL-34 (50 ng/mL Bio-techne) at 37 °C and 5% CO_2_ for 24 h. After incubation, cells were harvested, washed, and prepared for staining.

### 2.5. Flow Cytometry

Circulating monocytes (CD14+) expressing allograft inflammatory factor-1 (AIF-1; Iba-1) were identified following immunostaining of the whole PB-derived nucleated cell (NC) fraction. Erythrocytes were lysed using BD Pharm Lyse Buffer (BD Biosciences Pharmingen, San Diego, CA, USA). For flow cytometry analysis, 1 mL nucleated cells were resuspended in 100 μL of PBS. Immunofluorescence cell staining was performed with the use of the fluorescent conjugated antibody CD14-PE (BD Biosciences, San Jose, CA, USA). After 20 min incubation on ice, the cells were washed twice in PBS. Then, cells were fixed in 3.5% paraformaldehyde for 20 min, permeabilized using 0.1% Triton X-100 for 5 min, washed twice in PBS, and subsequently stained with anti-human Iba-1-FITC (Santa Cruz Biotechnology, Santa Cruz, CA, USA). After incubation for 1 h at room temperature, the cells were washed twice in PBS, resuspended, and analyzed using a NAVIOS Flow Cytometer (Beckman Coulter, Brea, CA, USA). Appropriate isotype control antibodies were used for each staining. The analysis was based on the characteristic marker of PB monocytes (CD14+) and Iba-1 expression. The cells had the concomitant presence of CD14 surface marker and intracellular expression of the Iba-1 marker. Kaluza software version 1.2 (Beckman Coulter, Brea, CA, USA) was used for the analysis. At least 106 events were acquired from each sample. The population of circulating AIF-1+/CD14+ cells was presented as the percentage of WBC, as detected by flow cytometry. 

### 2.6. Immunofluorescence Analysis of Synovial Samples

For immunofluorescence analysis, sections were deparaffinized in xylene and hydrated in solutions with decreasing ethanol concentrations (100–50%), followed by blocking in 10% normal donkey serum for 30 min at room temperature. To study colocalization of AIF-1 and CD68 proteins, sections were first incubated with the primary antibody, rabbit anti-AIF-1 (1:100) (Proteintech Group, Chicago, IL, USA), at 4 °C overnight. On the next day, the secondary antibody donkey anti-rabbit Alexa Fluor 647 (1:100) (Life Technologies, Carlsbad, CA, USA) was applied. Next, the incubation with primary antibody mouse anti-CD68 (1:200) (LifeSpan Biosciences, Seattle, WA, USA) and subsequent goat anti-mouse Alexa Fluor 488 F(ab’)2 (1:100) (Life Technologies, USA) was performed. Upon termination, the sections were counterstained with DAPI solution (Thermo Scientific, Pittsburgh, PA, USA), mounted in fluorescent mounting medium (Dako, Glostrup, Denmark), and subjected to microscopy analysis using an LSM700 confocal system (Carl Zeiss, Jena, Germany).

### 2.7. Statistical Analysis

Since we were analyzing a small number of samples, we applied non-parametric tests for the statistical analysis of AIF-1 expression. The Mann–Whitney *U*-test was used to compare expression between patient groups, and the Spearman rank correlation coefficient (*R*s) was used to study correlations between the expression of different AIF-1 isoforms in patient groups. Friedman ANOVA and Wilcoxon tests were performed to check the effect of stimulation of PBMCs by IL-6, IL-21, and IL-34 on the percentage of AIF-1-positive cells. A value of *p* < 0.05 was considered statistically significant.

## 3. Results

### 3.1. Expression of AIF-1 Isoforms in PBMCs and SMs in Patients with RA and OA

We detected three AIF-1 mRNA expression variants in PBMCs and SMs from both patient groups. In PBMC fractions, we detected the lowest expression in the v.4 transcript and highest expression in the v.1 transcript in both groups; however, there were no statistically significant differences between the patient groups. In SM samples, the mRNA expression for all AIF-1 variants was similar in both groups, but it was much lower when compared to AIF-1 variant mRNA expression in PBMCs (Table 1).

We also established SM/PBMC ratios for AIF-1 variant mRNA expression (Table 2); the ratios were below 1 (range, 0.22 ± 0.08–0.44 ± 0.26), thanks to the lower AIF-1 variant mRNA expression in SMs when compared to PBMCs. Similarly, they were not significantly different between the groups (Table 2).

### 3.2. Correlations of AIF-1 Variant mRNA Expression

Spearman’s rank correlation coefficient tests showed strong, positive, and significant correlations between different AIF-1 variant mRNA expression levels in PBMCs or SMs in both patient groups (Table 3).

### 3.3. Correlation of AIF-1 Variant mRNA Expression Between PBMCs and SMs in Patient Groups

Additionally, we examined the correlation of AIF-1 variant mRNA expression between PBMCs and SMs in patients with OA and RA. There were no statistically significant correlations in AIF-1 variant mRNA expression levels between PBMCs and SMs in the patient groups (Table 4).

### 3.4. Percentage of AIF-1 Positive Cells in Blood and SMs of Patients with RA and OA

**Figure 1:**
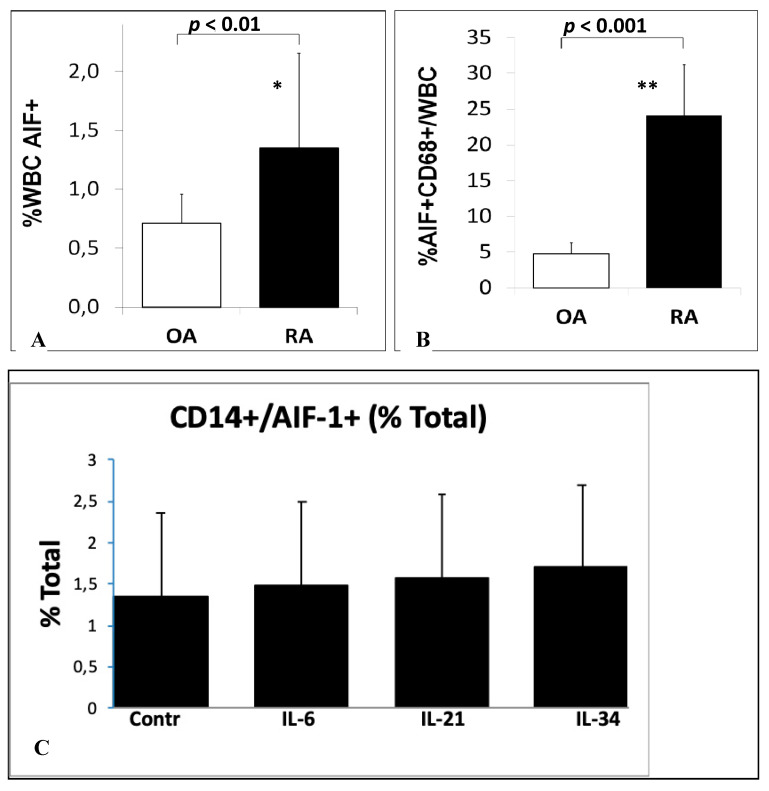
Flow cytometry analysis of WBC and PMBCs of OA and RA patients. (**A**) The percentage of WBC (white blood cell) AIF-1 (allograft inflammatory factor-1)-positive cells in the blood of rheumatoid arthritis (RA) and osteoarthritis (OA) patients, * *p* < 0.01. (**B**) The percentage of AIF-1+/CD68+/WBC cells in synovial membranes of OA and RA patients, ** *p* < 0.001. (**C** )CD14+/AIF-1+ cells as a % of total cell count after peripheral blood mononuclear cell (PBMC) stimulations; the differences were statistically non-significant.

**Figure 2:**
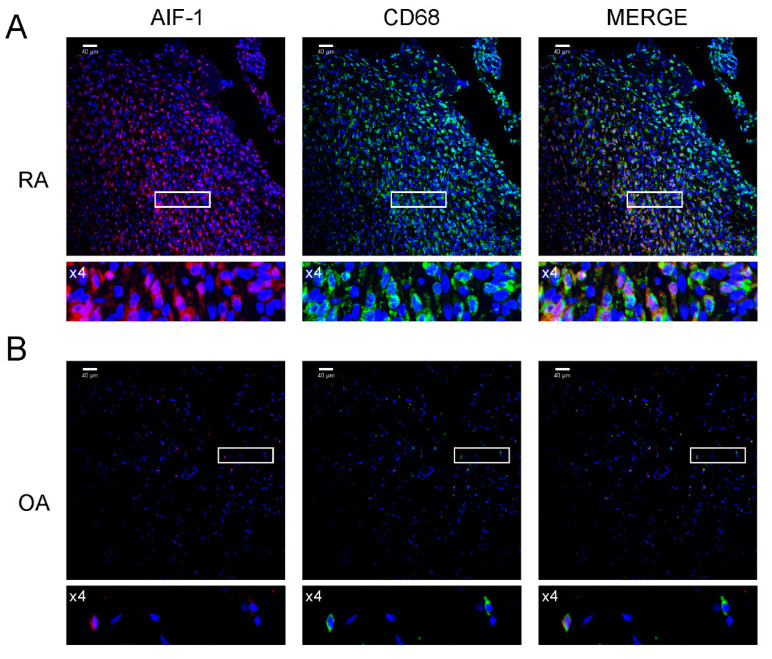
Immunolocalization of AIF-1 and CD68 in synovial membrane sections. Images are shown in separate fluorescence channels: red for AIF-1, green for CD68, as well as merged. (**A**) Sections from RA patients, showing predominant expression of AIF-1/CD68 cells compared to osteoarthritis patients. (**B**) Sections from OA patients. Four-fold magnifications of the highlighted area are included under each photo. The scale bar is 40 μm; only representative images are presented. Lower panels show regions of interest- ROI (white frames) with higher power magnification (zoom ×4).

We examined the percentage of AIF-1-positive cells in the blood of patients with RA and OA. The percentage of AIF-1-positive WBCs in patients with RA and OA was 1.35 ± 0.81% and 0.71 ± 0.25% (*p* < 0.01), respectively, whereas the percentage of AIF-1/CD68 cells in the SMs was 24.05 ± 7.17% and 4.78 ± 1.52% (*p* < 0.0001), respectively (Figure 1). We confirmed this result in histological samples of synovial membranes, were cells stained for AIF-1/CD68 were predominant on RA patients samples (Figure 2).

### 3.5. The Effect of IL-6, IL-21, and IL-34 Stimulation of PBMCs on the Percentage of AIF-1-Positive Cells

We examined the effect of stimulation of PBMCs by IL-6, IL-21, and IL-34 on the percentage of AIF-1-positive cells. After the stimulation of PBMCs by IL-6, IL-21, and IL-34, we observed 10%, 15%, and 27% increase in AIF-1-positive CD14 cells in stimulated PBMCs, respectively. The differences were statistically non-significant (Figure 1C).

## 4. Discussion

The aim of this study was to investigate if AIF-1 is the cytokine initiating the inflammatory process in RA or is produced under the influence of pro-inflammatory mediators involved in the induction and maintenance of the inflammatory status in patients with RA. Therefore, we examined the expression of AIF-1 isoforms in patients with RA and OA at the mRNA level, as well as measured the percentage of AIF-1-positive cells in the blood and SMs of patients with RA and OA. Our results showed a lack of statistically significant differences in the mRNA expression of AIF-1 isoforms between patients with RA and OA. However, the percentage of AIF-1-positive cells was significantly higher in patients with RA in comparison to patients with OA. This might have resulted from increased post-transcriptional activation of AIF-1 in patients with RA, or the mediators implicated in RA inflammatory processes might have induced the increased AIF-1 production in this patient group. A previous study has indicated that AIF-1 protein expression is induced by the pro-inflammatory cytokines IL-1β and tumor necrosis factor (TNF)-α, which are involved in the pathogenesis of RA [6]. In the present study, we examined the percentage of AIF-1-positive CD14 cells after stimulation by IL-6, IL-21, and IL-34. We observed a 10–27% increase in AIF-1-positive CD14 cells; however, the differences were statistically non-significant. The in vivo simultaneous action of many pro-inflammatory mediators probably caused an increase in AIF-1-positive cells.

So far, three AIF-1 splicing variants have been described in Uniprot.org (P55008-AIF-1_Human) [23]. The NCBI gene database shows only two (v.1 and v.3) AIF-1 isoforms, whereas AIF-1 v.4 codes for the same protein as v.1 [20]. In this study, we separately estimated mRNA expression levels for v.1 and v.4 to show differences in their expression profiles and possible correlations.

From the literature, AIF-1 v.1 and v.3 are well-characterized in patients with breast cancer; both are up-regulated in breast cancer adipose tissue (monocytes and macrophages) when compared to healthy controls [14]. In other work, AIF-1 v.3 has been up-regulated in PBMCs from cardiac transplant patients after reperfusion, suggesting that AIF-1 v.3 may be an innate immune response sensor in post-transplantation reactivity to allograft procedures [24]. We detected the mRNA expression of AIF-1 variant isoforms in PBMCs. This observation agreed with previous studies in patients with systemic sclerosis and patients with cardiac allograft rejection [21,24]. In PBMCs, the expression of AIF-1 variant isoforms was similar in patients with RA and OA, with higher expression levels for v.1 and v.3 when compared to v.4 in both patient groups. In SM cells, the mRNA expression for all variants was similar in both patient groups.

We observed that the mRNA expression of AIF-1 variant isoforms in PBMCs and SMs was correlated. These observations agreed with data from patients with breast cancer, where strong correlations between AIF-1 v.1 and v.3 were observed in PMBCs and breast cancer adipose tissue [14]. Variants in patients with breast cancer have also been correlated with body mass index, waist to hip ratio, and expression of COX-2, IL-6, and Cyp19A1 in breast adipose tissue [14].

We demonstrated higher AIF-1 variant mRNA expression in PBMCs when compared to SM cells. AIF-1 expression is not only described in monocytes/macrophages but also for some activated lymphocytes (T-cells) and blood neutrophils [4,8,25]. Blood cells may express more AIF-1 mRNA variants as they reflect general inflammatory mediator changes in patients with RA and OA when compared to local SM macrophages. We also established AIF-1 variant mRNA expression correlations between PBMCs and SMs in patients with RA and OA, but no statistical differences were observed. This observation did not agree with data from allograft transplantation studies, where AIF-1 expression in PBMCs was positively correlated with the prediction of allograft status or allograft rejection and was proposed as a biomarker for the detection of early allograft rejection [26,27,28].

In conclusion, AIF-1 mRNA expression variants occurred in PBMCs and SM cells in patients with RA and OA. AIF-1 mRNA expression levels were correlated with each other in PBMCs and SM cells, but there were no statistically significant correlations for AIF-1 mRNA expression variants between PBMCs and SMs in patients with RA and OA. Both in the blood and SMs, we observed an increased percentage of AIF-1-positive cells in patients with RA in comparison to patients with OA. The above results suggested that AIF-1 was the cytokine involved in the pathogenesis of RA. It seemed that AIF-1 was not the cytokine initiating the inflammatory process in RA. Probably the stimulation by various pro-inflammatory mediators, such as cytokines and chemokines, might increase AIF-1 expression in RA patients [6]. On the other hand, as previously shown, AIF-1 stimulated macrophages to produce pro-inflammatory cytokines, increasing inflammatory status in RA [6]. The precise knowledge of the role of AIF-1 in RA pathogenesis and the development of inflammatory status requires further investigations. 

## Figures and Tables

**Table 1:** mRNA expression for allograft inflammatory factor-1 (AIF-1) isoforms in PBMC and SMs in patients of osteoarthritis (OA) and rheumatoid arthritis (RA) groups.

Parameters		OA			RA			OA vs. RA
		*n*	Mean ± SD	Median (Q1–Q3)	*n*	Mean ± SD	Median (Q1–Q3)	*p* ^&^
PBMC	*AIF1v1*	12	4.23 ± 1.33	4.21 (3.38–4.81)	15	4.33 ± 1.30	4.06 (3.49–4.59)	1.00
	*AIF1v4*	12	2.60 ± 0.87	2.36 (2.12–3.42)	15	3.14 ± 1.10	2.80 (2.37–3.72)	0.20
	*AIF1v3*	12	3.72 ± 1.28	3.60 (2.82–4.87)	15	4.36 ± 1.22	3.88 (3.51–5.67)	0.16
SM	*AIF1v1*	12	1.00 ± 0.37	1.02 (0.73–1.32)	8	0.95 ± 0.31	0.90 (0.73–1.20)	0.76
	*AIF1v4*	12	1.00 ± 0.36	1.16 (0.69–1.26)	8	0.93 ± 0.26	0.82 (0.74–1.14)	0.70
	*AIF1v3*	12	1.00 ± 0.36	1.04 (0.72–1.27)	8	1.09 ± 0.35	0.99 (0.83–1.36)	0.82

^&^ Mann–Whitney *U*-test; PBMC: peripheral blood mononuclear cells; Q1: lower quartile; Q3: upper quartile; SD: standard deviation; SM: synovial membrane.

**Table 2:** SM/PBMC ratio for the expression of mRNA for AIF-1 isoforms in OA and RA groups of patients.

Parameters		OA			RA			OA vs. RA
		*n*	Mean ± SD	Median (Q1–Q3)	*n*	Mean ± SD	Median (Q1–Q3)	*p* ^&^
SM/PBMC	*AIF1v1*	12	0.27 ± 0.15	0.21 (0.16–0.36)	8	0.22 ± 0.08	0.22 (0.17–0.27)	0.76
	*AIF1v4*	12	0.44 ± 0.26	0.38 (0.28–0.56)	8	0.31 ± 0.10	0.33 (0.24–0.39)	0.25
	*AIF1v3*	12	0.32 ± 0.23	0.28 (0.19–0.36)	8	0.27 ± 0.09	0.27 (0.23–0.31)	0.94

^&^ Mann–Whitney U-test; PBMC: peripheral blood mononuclear cells; Q1: lower quartile; Q3: upper quartile; SD: standard deviation; SM: synovial membrane.

**Table 3:** Spearman’s rank correlation coefficient test for the mRNA expression of different AIF-1 isoforms in PBMC or SM in OA and RA groups of patients.

Parameters		OA				RA			
		*AIF1v4*		*AIF1v3*		*AIF1v4*		*AIF1v3*	
		R_s_	*p*	R_s_	*p*	R_s_	*p*	R_s_	*p*
PBMC	*AIF1v1*	0.82	0.0011	0.86	0.00033	0.91	0.000003	0.66	0.0078
	*AIF1v4*	-	-	0.94	0.000007	-	-	0.73	0.0021
SM	*AIF1v1*	0.76	0.0045	0.78	0.0030	0.95	0.00026	0.88	0.0039
	*AIF1v4*	-	-	0.83	0.0010	-	-	0.95	0.00026

R_S_: Spearman’s rank correlation coefficient; PBMC: peripheral blood mononuclear cells; SM: synovial membrane.

**Table 4:** Spearman’s rank correlation coefficient test for the mRNA expression of AIF-1 isoforms in PBMC and SM in OA and RA groups.

Parameters		OA						RA					
		PBMC						PBMC					
		*AIF1v1*		*AIF1v4*		*AIF1v3*		*AIF1v1*		*AIF1v4*		*AIF1v3*	
		R_s_	*p*	R_s_	*p*	R_s_	*p*	R_s_	*p*	R_s_	*p*	R_s_	*p*
SM	*AIF1v1*	−0.05	0.88	−0.23	0.47	−0.12	0.71	0.52	0.18	0.31	0.46	0.55	0.16
	*AIF1v4*	0.09	0.78	−0.12	0.71	0.10	0.75	0.40	0.32	0.14	0.74	0.33	0.42
	*AIF1v3*	0.04	0.90	−0.13	0.70	0.08	0.80	0.26	0.53	0.10	0.82	0.12	0.78

R_S_: Spearman’s rank correlation coefficient; PBMC: peripheral blood mononuclear cells; SM: synovial membrane.
